# Cell lineage tracing in the developing enteric nervous system: superstars revealed by experiment and simulation

**DOI:** 10.1098/rsif.2013.0815

**Published:** 2014-04-06

**Authors:** Bevan L. Cheeseman, Dongcheng Zhang, Benjamin J. Binder, Donald F. Newgreen, Kerry A. Landman

**Affiliations:** 1 Department of Mathematics and Statistics, University of Melbourne, Parkville, Victoria 3010, Australia; 2 Murdoch Childrens Research Institute, Royal Children's Hospital, Parkville, Victoria 3052, Australia; 3 School of Mathematical Sciences, University of Adelaide, Adelaide, South Australia 5005, Australia

**Keywords:** cell lineage, invasion wave, enteric nervous system, cell-fate decisions

## Abstract

Cell lineage tracing is a powerful tool for understanding how proliferation and differentiation of individual cells contribute to population behaviour. In the developing enteric nervous system (ENS), enteric neural crest (ENC) cells move and undergo massive population expansion by cell division within self-growing mesenchymal tissue. We show that single ENC cells labelled to follow clonality in the intestine reveal extraordinary and unpredictable variation in number and position of descendant cells, even though ENS development is highly predictable at the population level. We use an agent-based model to simulate ENC colonization and obtain agent lineage tracing data, which we analyse using econometric data analysis tools. In all realizations, a small proportion of identical initial agents accounts for a substantial proportion of the total final agent population. We term these individuals superstars. Their existence is consistent across individual realizations and is robust to changes in model parameters. This inequality of outcome is amplified at elevated proliferation rate. The experiments and model suggest that stochastic competition for resources is an important concept when understanding biological processes which feature high levels of cell proliferation. The results have implications for cell-fate processes in the ENS.

## 1. Introduction

Lineage tracing is a powerful tool for understanding how cells behave within a biological process, where a single cell is marked (labelled), and this mark is inherited by all progeny. The contribution of an individual cell lineage can then be traced within a population of cells [1]. Recent advances in experimental techniques [2–5], light microscopy and image processing [6–10] have increased the scope and potential use of lineage tracing methods to address important developmental questions. For example, are cell-fate decisions hard-wired or environmentally based, and how do cell-fate decisions result in the morphological and cytotypic development of complex tissues?

Cell lineage tracing combined with mathematical modelling has been implemented to understand the interaction between individual cell decisions and the overall response in the immune system [11,12]. These authors study a model of intracellular stochastic competition of cell decisions that is able to capture experimental immune response dynamics. However, the model has no significant spatial component such as is seen in solid tissues, where balancing of cell differentiation options must be appropriate not only at the scale of the entire population, but also at the mesoscale of each spatial domain occupied by cells.

In developmental processes, cell movement and division can be affected by availability and organization of embryonic tissue space, which is not static but growing. Similar arguments can be made for other spatially distributed microenvironmental requirements such as growth factors. Therefore, the impact of spatial components on individual cell dynamics and resulting cell lineages needs to be determined. Here, we explore tracing cell lineage in a colonization process through experiments and simulation of a fundamental developmental system.

Enteric neural crest (ENC) cells migrate from the vagal neural crest (NC) to the foregut, and then progress from the foregut as a strictly timetabled rostrocaudal wave to colonize the whole of the gastrointestinal tract, forming the enteric nervous system (ENS). The cell density of the mature ENS and the proportions of neuronal and glial cell types are consistent between individuals [13]. Although nearly all the ENS is derived from only three to four segment lengths of NC, the ENS rivals the spinal cord in neuron number, and has numerous neuron types. This requires massive and controlled proliferation and differentiation unprecedented in the peripheral nervous system. For example, in quail embryos starting with about 1000 ENC cells [14], the number increases to 350 000 over 5 days. During the development of the ENS, the ENC cell population moves and proliferates in surrounding mesenchymal (gut) tissue, which is simultaneously elongating through cell division [15].

In both non-growing and growing gut tissues, we perform organ culture experiments that examine ENC cell colonization by labelling a single cell in the starting population and counting the cell's progeny at a later time. At the population level, the ENS is highly organized, and one might assume that to produce such a predictable pattern with constancy of neural distribution, numbers, density and type proportions would require deterministic processes. By contrast, we show that cell lineage tracing reveals an extremely large variability in the number and distribution of progeny of single founder ENC cells. This variability is further examined using an agent-based or cellular automata (CA) model.

Over recent years, ENC migration has been simulated on both a non-growing and growing domain (gut tissue) [14–18]. Each agent represents an ENC cell, and agent movement and proliferation are determined stochastically, as claimed in this biological process. The total agent population was found to be highly predictable. However, we have observed that individual agent contributions can be highly variable theoretically [19], although this was not quantified nor its basis explored. These paradoxical findings provide the motivation for the modelling and quantitative analysis of agent lineage tracing and comparison with new ENC cell lineage tracing data, presented here for the first time.

Econometric data analysis tools, Lorenz curves and Gini coefficients, are adapted to analyse the agent lineage tracing data [20–25]. The *in silico* frequency distributions of clonal contributions (number of progeny agents derived from a single agent) exhibit a large proportion of agents having very few progeny and a very small proportion of agents contributing a significant proportion of the total population. When analysed at an individual realization (*in silico* experiment) level, a consistent and persistent dynamic in the lineage tracings emerges. We find that in all realizations a small proportion of otherwise identical initial agents accounts for a substantial proportion of the total population of agents. We term these individuals superstars. We show that the existence of a few superstars is consistent across individual realizations, and is robust to changes of the model parameters (e.g. domain growth, no domain growth, initial agent density). Therefore, *in silico*, clonal dominance is an emergent property of a homogeneous starting population subjected to stochastic motility and proliferation rules.

The ENC lineage tracing implies that self-organization principles of ENS development are predictable at the population level, but show stochastic diversity at the level of individual cells. Comparison of our experimental data with our agent-based model results suggests that clonal dominance through stochastic competition is a fundamental feature in the creation of the ENS. Our findings also have important implications for cell-fate processes. In particular, the results suggest that cell differentiation occurs after colonization, as stochastic competition for resources would permit early fate decisions to lead to highly unpredictable cell-fate distributions. The methods can be generalized to other biological processes during and after development, such as tumour invasion, because stochastic competition for resources (e.g. space, growth factor, nutrient) is fundamental to a proliferating invading cell population.

## 2. Material and methods

### 2.1. Experimental methods

**Figure 1.**
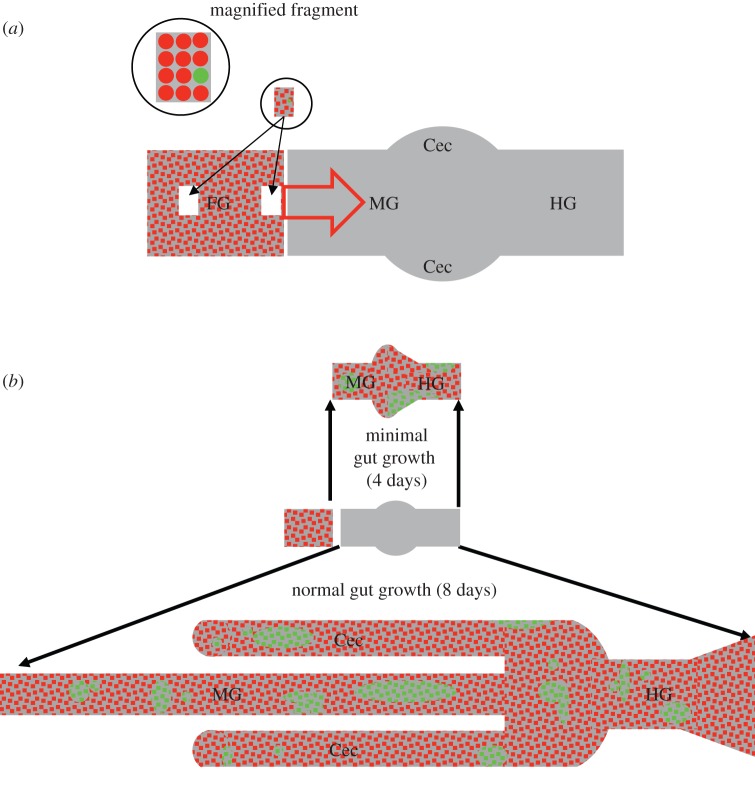
Schematic diagram of the ENS experiments. (*a*) Diagram of culture system set-up. Quail E4 foregut (FG) with ENC cells (red (mid-grey) dots) is supplemented with a fragment of E3.5 FG with one GFP positive ENC cell (green (light grey) dot). This is placed either centrally or at the distal edge of the FG. This moiety is fused to an E4 post-umbilical midgut (MG) and hindgut (HG) with bilateral cecae (Cec). This gut region is uncolonized by ENC cells. Colonization then proceeds in an MG to HG wave of ENC cells (large red arrow). (*b*) The initial set-up is grown either for 4 days as an organ culture *in vitro*, where there is minimal tissue growth, or for 8 days as a CAM graft where the gut grows similarly to normal. The uncolonized MG and HG becomes occupied by ENC cells (red (mid-grey) dots) including clonal derivatives of the original GFP positive ENC cell. These GFP positive derivatives show unpredictable numbers and distributions at the end of the growth period. (Online version in colour.)

Quail embryo pre-migratory vagal NC cells are electroporated with a genome-integrating green fluorescent protein (GFP) plasmid at embryonic age day 1.5 (E1.5), as in Binder *et al.* [16]. Vagal NC cells migrate to the foregut by E3 at which point they acquire the ability to colonize gut mesoderm and are referred to as ENC cells [26]. At E3.5, a fragment of foregut (typically 10^−3^ mm^3^) containing one GFP positive ENC cell is isolated by microdissection and combined with an E4 foregut containing an entire unlabelled GFP negative ENC cell population (approx. 8000 cells). The foregut at E3.5–4 is about diameter of 0.3–0.5 mm and length of 1–1.5 mm. This provides a normal quota of ENC cells for further gut colonization, with one cell labelled with a marker detectable in all progeny of that cell. These tissues are placed in line with, and rapidly fuse to, the rostral end of a chick E4 aneural mid- and hindgut to permit a rostrocaudal colonization wave of ENC cells [27]. In some cases, the fragment bearing the GFP positive ENC cell is sandwiched between the foregut and the aneural gut, so the GFP positive cell is positioned at the ENC cell wavefront. In other cases, the foregut is cut into two, and the GFP positive cell fragment is sandwiched between these halves, placing the GFP positive cell nominally about 500 μm behind the wavefront. Figure 1*a* illustrates the experimental set-up. Owing to frontal expansion, a cell's proximity to the wavefront is known to favour further contribution to the colonizing wave [27,28]. The combination is grown in catenary culture *in vitro* for 4 days (minimal gut growth; [29]) or 8 days in a chorio–allantoic membrane (CAM) graft (near normal gut growth; [14]) to allow colonization of the midgut, caecum and hindgut, as shown in figure 1*b*. In the latter case, the gut is dissected free of the CAM membranes to display the complex intestinal morphology.

ENC cell derivatives, both GFP positive and GFP negative, colonize the recipient gut as two layers (myenteric and submucosal plexuses). Cell types are identified as neurons or glial/ENC cell types by immunolabelling gut wholemounts for Hu and SoxE, respectively, as in [16]. Olympus IX70 microscope (Olympus Optical Co., Tokyo, Japan), spot monochrome camera 2.1.1, Image Pro Plus 4.5 and Image Pro Analyser 6.1 (MediaCybernetics, Silver Spring, MD, USA) are used for microscopy, imaging and analyses. Confocal images are acquired with an Leica CLSP confocal microscope. Cell counts in the CAM grafts include only those GFP positive cells that had moved into the recipient midgut and hindgut. In one case (figure 3*b*) where GFP positive cell numbers are extremely large but seemingly uniformly distributed, GFP positive cell numbers are estimated by counting cells in five squares of 10^−2^ mm^2^ and estimating the total area of the myenteric plexus, assuming the intestinal tubes were cylindrical. Note that this underestimates the actual cell number, because the deeper and smaller submucosal plexus is not included.

### 2.2. Cellular automata model

A discrete-time, agent-based CA model on a regular lattice is used. This model is explained in further detail in Binder & Landman [18]. Here, a square lattice is used (the choice of lattice is not important here—a triangular lattice has also been used for other ENS applications [30]). A two-dimensional rectangular domain of length *L* and width *Y* is appropriate, because the ENC cells are restricted to a cylindrical surface within the intestinal tissue. For the case of an elongating gut tissue, the length of the domain (i.e. the lattice) increases exponentially in time *t* with growth rate *α* (to match experimental findings [15]), through random insertion of new lattice sites [15,18]. This provides the framework to simulate the ENS colonization, with a single agent (representing an ENC cell) occupying a single lattice site at any time *t*. We simulate the two main mechanisms in the colonization process, ENC cell motility and proliferation [18,31]. (No agent death is included here, because there is little evidence of ENC cell death during the colonization process [32]. Agent differentiation from ENC agent to neuronal agent is not explicitly included here, but could be included with a reduced proliferation rate [17].)

During a time step from *t* to *t* + 1, an asynchronous updating scheme is used. The *m*(*t*) agents are selected uniformly at random and given the opportunity to move and then to proliferate. For a motility event, a chosen agent at (*x*, *y*) attempts to move with probability *P*_m_ to one of the four nearest neighbours (*x* ± 1, *y* ± 1) with equal probability (1/4). For a proliferation event, a mother agent at (*x*, *y*) attempts to divide with probability *P*_p_, and one daughter remains at (*x*, *y*), whereas the second daughter is placed at either (*x* ± 1, *y*) or (*x*, *y* ± 1) each with equal probability (1/4). (Note that this proliferation rule places daughters adjacent to each other, as in Binder *et al.* [16], rather than be separated by a single site, as in Binder & Landman [18]. Both rules give qualitatively similar results.) If the target site is occupied for any motility or proliferation event, then the event is aborted. Such a process is called an exclusion process [33]. To represent the cylindrical geometry of the intestine, periodic boundary conditions along the horizontal boundaries and no-flux boundary conditions along vertical boundaries are imposed. The initial condition is taken to be 10 fully occupied left-most columns of the lattice. Different initial conditions and different agent carrying capacities were considered with no qualitative change in the results (see the electronic supplementary material).

Parameter values are chosen to be consistent with the ENC colonization process. A single lattice spacing represents a cell diameter (10 μm), and the lattice width (*Y* = 50) represents the midgut ENS plexus layer circumference (≈500 μm). Alternative gut widths show no qualitative change in results (so our results are not an artefact of the boundary conditions; see the electronic supplementary material). The gut length is greater than 500 μm at E3.5. The simulation time step represents approximately 15 min, representing the average time for a cell to move one cell diameter (average speed: 40 μm h^−1^ [34,35]). Because cells move approximately one lattice spacing every time step, we set *P*_m_ = 1. We investigate the effect of varying *P*_p_. Without loss of generality, other representative length and timescales could be used. However, the ratio *P*_p_/*P*_m_ drives the dynamics, and a change in *P*_m_ is analogous to a change in length or timescales. The range of values for the domain growth rate *α* spans the growth rates obtained experimentally for quail embryos [15].

**Figure 2.**
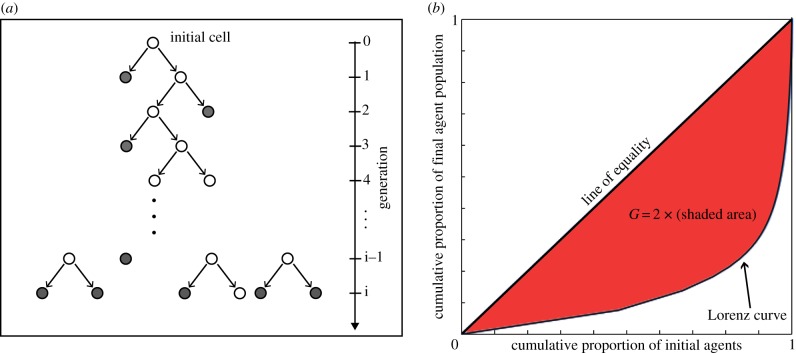
Schematic lineage tracing diagram and a Lorenz curve. (*a*) Lineage of a single agent at time *t*. An empty circle represents an agent division event. Only the grey-filled circles are counted to determine the agent lineage. (*b*) Lorenz curve and Gini coefficient *G*, which is twice the shaded region. (Online version in colour.)

To obtain agent lineage tracings, each initial agent is uniquely marked and all its progeny retains the same unique marker. We define the lineage tracing at a particular time *t* to be the total number of agents descended from the original agent present at *t* = 0, as illustrated with the grey (filled disc) agents in figure 2*a*. Clearly, the distribution of lineage tracings is a function of the total number of agents and consequently the length of the invasion wave. The time to reach any particular length is determined by the speed of the invasion wave, which depends (in a nonlinear way) on the probability of proliferation *P*_p_ (fixing *P*_m_ = 1) [31,36]. To independently assess the impact of the proliferation rate on the clone distribution, rather than on the total number of agents, the total agent population is fixed instead of the elapsed time for the simulation. Further, this reduces the variability in the migration length (instead introducing variability in elapsed simulation time) that occurs when terminating the simulations at some elapsed fixed time. The relationship between the mean total agent number versus elapsed time for various proliferation rates is provided in the electronic supplementary material.

### 2.3. Quantification tools for lineage distributions

The agent lineage simulation data have a consistent subpopulation whose contribution to the total population is significantly larger than the contribution of the majority of the population. This results in a distribution with a long tail. The focus here is not to classify the exact properties of these distributions, but rather to determine their qualitative features and to compare their behaviour across independent samples. In particular, we are interested in comparing the cumulative contribution of individuals to the total final population.

Two measures for investigating such distributions are the Lorenz curve [20,25] and Gini coefficient [21], arising in the study of economic data such as income distributions [22]. The Lorenz curve gives insights into how evenly a measure, such as wealth or size of lineage distributions, is distributed across a population with respect to the total pool of this measure (e.g. wealth or total cell or agent population), represented in figure 2*b*. Our Lorenz curves are defined in the following way.

For a single simulation with an initial agent population size *n*, we define the sequence *x_j_* (*j* = 1, 2, *…* , *n*) to be the ranked non-decreasing lineage tracings, giving *x*_1_ ≤ *x*_2_ ≤ *…* ≤ *x_n_*. (Note that the original agent is counted in the tracings—an agent with no progeny has its lineage tracing equal to unity.) The Lorenz curve [23,24] is the polygon joining the points (*h*/*n*, *L_h_*/*L_n_*), for *h* = 0, 1, 2, *…* , *n*, where2.1
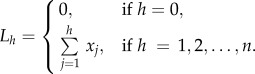


The line of equality (figure 2*b*) corresponds to a perfectly equal number of progeny from each initial agent; hence, any proportion of initial agents, say *p*, always has proportion *p* of the total agent population. For unequal distributions, the Lorenz curve tells us how much of a total measure (e.g. total agent population) is accounted for by a set proportion of the ‘poorest’ individuals in the population, or conversely the proportion accounted for by a set proportion of the ‘richest’ individuals.

The Lorenz curves are constructed by tracing each starting agent in 200 simulations. These are almost indistinguishable from Lorenz curves constructed by tracing every starting agent in just a single simulation.

Lorenz curves for each column are also determined, where only agents originating in a particular column are considered. Let *i* = 0 correspond to the right-most column and *i* = −1, −2, … −9 correspond to agents at position *i* from the right-most column (starting in general with 10 columns; figure 4*a*). Only agents from the lineage of agents originating in the *i*th column are used in the calculation of the Lorenz curve corresponding to the *i*th column. Hence, the *i*th column Lorenz curve shows the cumulative sum of the ordered contributions of the *i*th column agents. For a single simulation with *n_i_* initial agents in column *i*, we define the sequence *x*(*i*)*_j_*, (*j* = 1, 2, *…* , *n_i_*) to be the ranked non-decreasing lineage tracings, giving *x*(*i*)_1_ ≤ *x*(*i*)_2_ … ≤ *x*(*i*)*_n_i__*. Then, the column *i* Lorenz curve is the polygon joining the points (*h_i_*/*n_i_*, *L*(*i*)*_h_*/*L*(*i*)*_n_i__*), for *h_i_* = 1, 2, *… n_i_*, where2.2
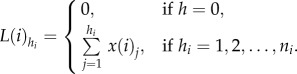


The Gini coefficient is used to analyse the time evolution of the inequality in the Lorenz curves [21]. Given the ranked agent tracing data *x_j_* discussed above, the Gini coefficient *G* is calculated as [24]2.3
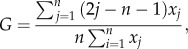
where 0 ≤ *G* < 1 (figure 2*b*). A low Gini coefficient indicates a more equal distribution, with *G* = 0 corresponding to complete equality, whereas a higher Gini coefficient indicates a more unequal distribution.

We determine the ordered lineage tracings *x_j_*(*n*) as a function of the total agent population *n*, or alternatively, *x_j_*(*t*), the ordered lineage tracings at a particular time *t*.

## 3. Results

### 3.1. Experimental results

The data for the cell lineage numbers (GFP positive cells) from individual non-growing and growing gut tissue experiments are presented in tables 1 and 2, where the gut is fully colonized. The 4 day catenary culture experiments allow minimal gut growth conditions (table 1), whereas the 8 day CAM grafts permit massive gut growth equivalent to normal growth owing to the presence of a blood supply (table 2).

**Table 1.** Four day catenary culture experiments with no gut growth (24 explants). In each a single GFP ENC cell was placed at or behind the wavefront.

cell count	placement of GFP cell	
	at wavefront, number of explants	behind wavefront, number of explants
0–9	6	5
10–19	1	1
20–29	0	1
30–39	1	0
40–49	1	2
50–59	1	0
60–69	1	1
70–79	1	0
80–89	0	0
90–99	2	0
>100	0	0

**Table 2.** Eight day CAM graft experiments with gut growth (62 grafts). In each a single GFP ENC cell was placed at or behind the wavefront. Note the uneven cell count intervals.

cell count	placement of GFP cell	
	at wavefront, number of explants	behind wavefront, number of explants
0–99	24	18
100–199	1	0
200–299	0	0
300–399	0	0
400–499	1	0
500–599	2	0
600–699	1	0
700–799	2	0
800–899	1	1
900–999	1	0
1000–1999	0	4
2000–2999	3	1
3000–3999	0	0
4000–4999	1	0
5000–20 000	0	0
>20 000	1	0

The cell lineage numbers are much smaller for the non-growing experiments, because both the final time and physical space that the ENC cells colonize is smaller than in the growing gut experiments. In general, the cell counts are larger when derived from a labelled cell placed at the wavefront than that from a cell placed behind the wavefront. We found experimental evidence of a superstar, with one cell lineage tracing (progeny) being of disproportionately huge number. Additionally, the probability of not migrating into the initially aneural recipient gut is elevated (i.e. count is zero) when starting behind the wavefront. However, some individual grafts still showed relatively large numbers of GFP positive cells.

**Figure 3.**
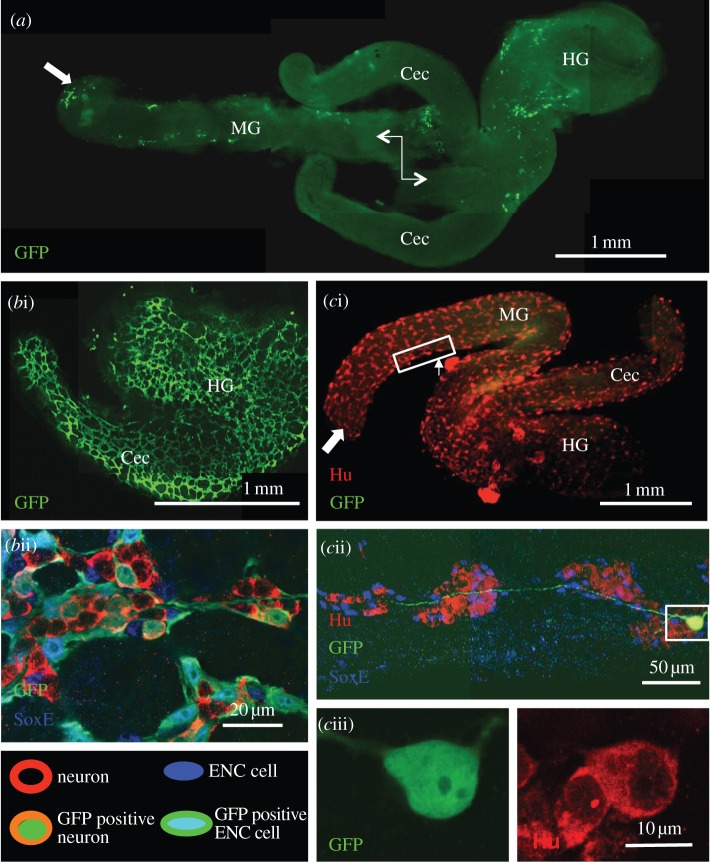
Normal gut growth. Grafts of midgut (MG), cecae (Cec) and hindgut (HG) after 8 days growth on CAM, with descendants of a single GFP positive ENC cell shown in green. (*a*) Specimen in which GFP positive cells are scattered along the intestine, from the original donor position in the rostral MG, indicated by broad arrow, to the HG. (*b*(i)) Confocal views of a superstar specimen with huge numbers of GFP positive cells densely forming the myenteric plexus throughout the intestine. (*b*(ii)) At higher magnification, triple labelling shows that the ENS ganglia contain both neurons (Hu label, red) and glial/ENC cells (SoxE label, blue), with GFP positive and negative cells in both categories. (*c*(i)) Specimen with only one GFP positive cell. This cell occurred in the MG (thin arrow), far displaced from the original donor position (broad arrow). Note that the entire gut is colonized, shown by ENS ganglia (Hu label). (*c*(ii)) Enlargement of the boxed area in (*c*(i)) shows this GFP positive cell extended a long axon rostrally through other ENS ganglia. (*c*(iii)) Enlargement of the boxed area in (*c*(ii)) confirms that this GFP positive cell is a neuron, with strong Hu labelling (red).

The GFP positive progeny or cell lineage tracings formed multiple loose patches of both ENC cells and neurons mixed with GFP negative ENS cells. The number and spatial distribution of GFP positive cells are completely unpredictable and variable, although the ENS viewed as a whole is always highly uniform. Figure 3*a* illustrates the large spatial extent of neural progeny. Figure 3*b* illustrates the progeny of the superstar (with more than 20 000 progeny shown in table 2). In this experiment, the descendants of this one superstar accounted for about one-third of all ENS cells. Figure 3*c* shows an example where there was only one labelled cell after 8 days, and this single cell had no progeny. This one cell, located at a distance from the original site, is a neuron with a prominent axon.

### 3.2. Model results

The simulations are terminated when the total agent population evolves to a fixed number of 6524. The target agent number occurs at a much earlier time for the growing domain than for the non-growing domain, because fewer proliferation events are aborted owing to the addition of extra lattice sites throughout the wave. In §3.2.3, only the non-growing case is discussed, because the growing case shows the same principle characteristics.

#### 3.2.1. Quantifying agent lineage tracings

**Figure 4.**
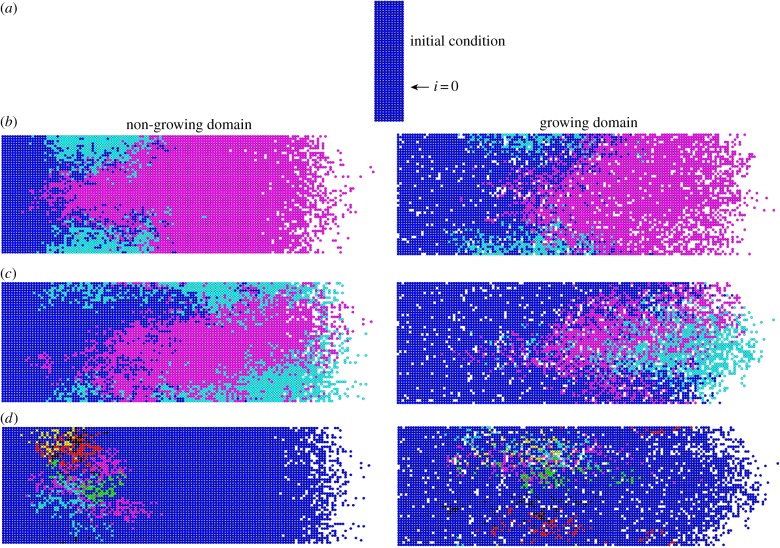
Spatial distribution of agent tracings for non-growing and growing (*α* = 0.003) domain simulations, *P*_p_ = 0.05. (*a*) Initial condition for all simulations. Each fully occupied column has an index *i*, where *i* = 0 corresponds to the right-most column. (*b*,*c*) Largest lineage tracing (pink), second largest lineage tracing (turquoise) and remaining agent population (blue). (*b*) Significant differences in the agent numbers between the two largest tracings. (*c*) The two largest tracings have a similar number of agent numbers. (*d*) The fifth to tenth largest tracings in (*c*). The total number of agents in (*b–d*) is the same. For the non-growing case this occurs at *t* ≈ 600, whereas for the growing case, *t* ≈ 400.

Starting with a small number of agents at the left end of the lattice (figure 4*a*), the agent colonization evolves and moves progressively to the right for both the non-growing and growing domain cases (figure 4). For the non-growing domain, the column-averaged lattice-site occupancy averaged over many identically prepared realizations evolves to a travelling density wave which moves from left to right with a constant speed, dependent on the probabilities associated with agent motility and proliferation [31]. Discrete-time mean-field arguments and continuum limits for the agent-based probabilistic model produce a partial differential equation for the average occupancy [37]. This equation is the well-known Fisher equation [38], which exhibits travelling wave solutions moving with a constant speed. The Fisher wave speed matches the mean wave speed in the agent-based model when the proliferation rate is low (relative to motility). Corrected mean-field arguments can be used for large proliferation rates [36,39]. When the domain grows, the wave no longer moves linearly with time [40,41].

Realizations with the largest (pink) and second largest (turquoise) lineage tracing, together with all progeny from the remaining initial agents (blue) are illustrated in figure 4*b*,*c*. The progeny of two superstars is evident, where combining the largest two tracings accounts for roughly the same proportion of the total population. However, figure 4*c* shows an almost equal split between the two tracings, unlike that seen in figure 4*b*. As time increases, the colonization in figure 4*b* continues to evolve from only one lineage tracing (pink), whereas in figure 4*c*, it will arise by stochastic competition from the two largest superstars (see the electronic supplementary material, video S1). Note that there is a larger proportion of non-superstar blue agents in figure 4*b,c* for the growing domain case, because additional space is continually being made behind the wavefront, allowing agents to proliferate in that region [19].

By way of contrast, the fifth to tenth largest contributors to the total population are shown in figure 4*d*. Their total numbers are modest and they lie well behind the wavefront. These agent tracings are stretched longitudinally, similar to many GFP positive cell assemblies in our experiments.

**Figure 5.**
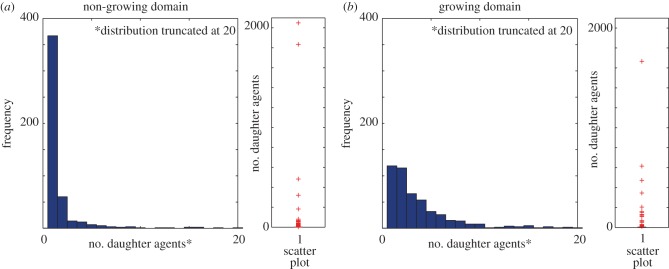
Single realization frequency distribution and scatter plot (500 data points), *P*_p_ = 0.05. (*a*) Non-growing domain, *α* = 0. (*b*) Growing domain, *α* = 0.003. Note the distributions in the histograms are truncated. (Online version in colour.)

To analyse the agent lineage tracings, we begin with examining the frequency distributions and scatter plots (showing the huge spread in the 500 data points) of a single realization for a non-growing and growing domain (figure 5). They are characterized by a large proportion of agents with low contributions, a long tail and the presence of superstars. However, for the non-growing domain case, there are a large number of agents that do not proliferate at all, and in the growing domain case, there is a far greater proportion of agents having between two and 15 progeny. This is expected, because additional space is continually being made in the growing domain simulations, allowing agents to proliferate in the already colonized part of the domain [19]. (Note that these histograms when plotted using a *log* scale also show the same features, and therefore do not help with the analysis.)

A Lorenz curve is a useful tool to quantify the distributions where inequality is a feature. We determine the Lorenz curves for the agent lineage data and investigate their dependence on agent proliferation rate and domain growth rate.

**Figure 6.**
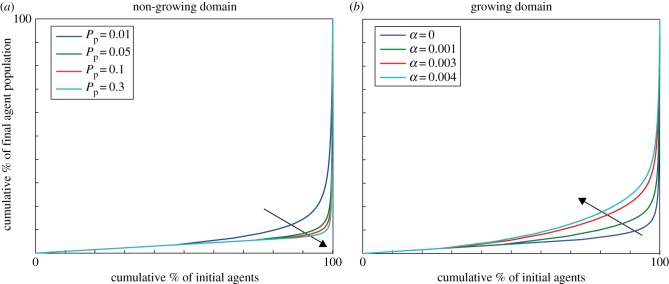
Lorenz curves. (*a*) Non-growing domain, *α* = 0. The arrow indicates increasing proliferation probability *P*_p_. (*b*) Growing domain, with *P*_p_ = 0.05. The arrow indicates increasing domain growth rate *α*. The curves show that a small proportion of the initial starting population is contributing to bulk of the total agent population. (Online version in colour.)

For all proliferation rates, the final population is dominated by the progeny of superstars, indicated by the sharp curvature in the distribution at high cumulative proportions in figure 6*a*. As the proliferation rate increases (relative to motility), the distribution of agent traces becomes more unequal (although the decrease in equality appears to occur at a diminishing rate, with the Lorenz curves converging). By contrast, as the domain growth rate increases, the bulk of the population contributes a larger percentage to the final agent population. This observation is reflected in the Lorenz curves for increasing values of *α* given in figure 6*b*. The Lorenz curves change over a broad range of the proportion of initial agents. Although we provide Lorenz curves obtained over 200 simulations here, the Lorenz curves from a single simulation are largely indistinguishable from the ones shown.

We note that in fixed elapsed time (as opposed to total agent number shown here) simulations, the growing domain case has a much larger final population of agents, resulting in a substantially enhanced agent progeny potential relative to the non-growing domain case. Additionally, we see that the proportion of agents that contribute to the final agent population increases as the exponential domain growth rate *α* increases.

**Figure 7.**
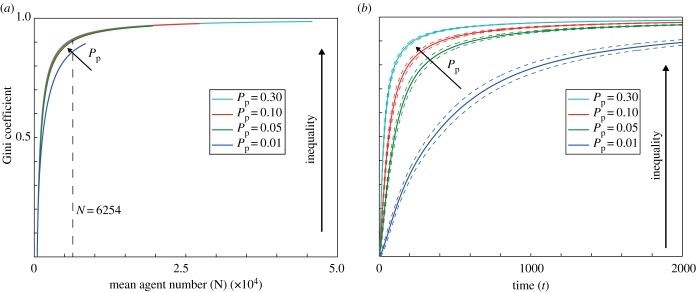
Gini coefficient for non-growing domain and various proliferation rates *P*_p_. (*a*) As a function of average total agent number. The vertical dashed line indicates the total agent number used in the Lorenz curves of figure 6*a*. (*b*) As a function of time (solid lines). The dashed lines indicate the 95% confidence intervals (±1.95*σ*). (Online version in colour.)

For the non-growing case, we calculate the corresponding Gini coefficients (figure 7*a*). The Gini coefficient increases as the average total agent number increases, and hence show that the lineage tracings become more unequal. However, it does not vary with proliferation rate significantly (except for *P*_p_ = 0.01), as also noted for the Lorenz curves. Alternatively, the Gini coefficient monotonically increases with time (figure 7*b*). For each time point, the Gini coefficient increases as the proliferation rate increases. When the proliferation rate is sufficiently small, there is a gradual increase towards high inequality with time, but when the proliferation rate is sufficiently large, there is a sharp increase towards high levels of inequality within a short time. From figure 7*a*, we deduce that the differences observed in figure 7*b* are largely owing to the variance in the total agent number in the case of the fixed elapsed time analysis.

The 95% confidence intervals for the Gini coefficient suggest that lineage tracings across simulations are consistently unequal and that inequality is an essential feature of all simulations. (The normality assumption appears to be valid here owing to the individual Gini indexes being independent and bounded.) This is also consistent with the fact that the Lorenz curves obtained from either a single simulation or from 200 simulations are largely indistinguishable. The variance of the Gini coefficient decreases with increasing proliferation rates, indicating that the process becomes more consistent in terms of its inequality as the proliferation rate increases.

In summary, the agent lineage tracing distributions are characterized by consistent large inequality. This inequality increases with both proliferation rate and total agent number (and as a proxy time), and decreases as the domain growth decreases.

#### 3.2.2. Superstars are present across individual realizations

Lorenz curves and Gini coefficients of data from 200 simulations show that the invasion process is characterized by a few superstars contributing to a large proportion of the population. To ascertain whether this is a feature of each simulation, we examine the percentage of agents in each simulation that account for a fixed percentage of the final population, as the proliferation rate and the domain growth rate are varied.

**Figure 8.**
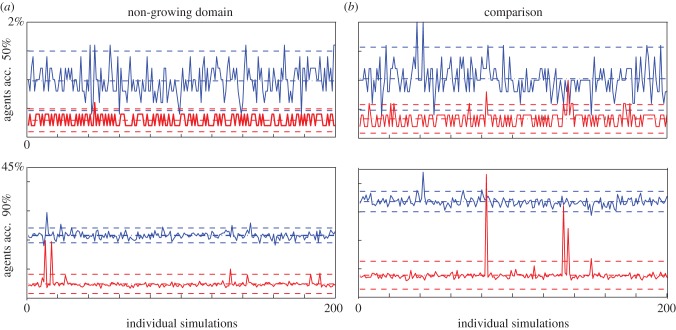
Consistency of superstars across 200 individual realizations. Smallest % of agent tracings required to account for 50% (top panels) and 90% (bottom panels) of final agent population. The mean and 95% confidence interval (±1.95*σ*) are shown with dashed lines. (*a*) Non-growing domain for two values of the probability of proliferation *P*_p_ = 0.01 (blue upper curve) and *P*_p_ = 0.1 (red lower curve). (*b*) Comparison between growing (blue upper curve, *α* = 0.003) and non-growing domains with *P*_p_ = 0.05 (red lower curve). (Online version in colour.)

In the non-growing domain case (figure 8*a*), a low proliferation rate (*P*_p_ = 0.01), 1% of the original population accounts for 50% of the final population, whereas for a larger proliferation rate (*P*_p_ = 0.1), 0.25% of the original population accounts for 50% of the final population. In addition, the data variance is small; for example, when *P*_p_ = 0.01, between two and eight agents account for 50% of the population. As expected, the percentage of agents required to account for 90% of the final population is greater, but again shows surprisingly small variance, with the exception of two small peaks for the higher proliferation case.

When comparing the growing and non-growing domain results, figure 8*b*, the percentage accounting for a fixed percentage of the final numbers increases markedly for the growing domain. This again emphasizes that a larger number of agents are contributing the bulk of the population.

This analysis illustrates that the existence of superstars is a ubiquitous feature of the invasion process.

#### 3.2.3. Superstars are not an artefact of agent starting position

In the analysis so far, all initial agents have been treated as equal, regardless of their starting position. However, agents initially in the right-most column are more likely to be able to proliferate than those in the remaining nine columns to the left. Because a single starting agent can account for half the population (figure 8*a*), we know that the starting position will not be the only factor in determining the presence of inequality. Indeed, there are 50 initial agents in the right-most column (and all agents within a column are equivalent owing to the boundary conditions), but at most one agent from the right-most column accounts for over 50% of the population.

**Figure 9.**
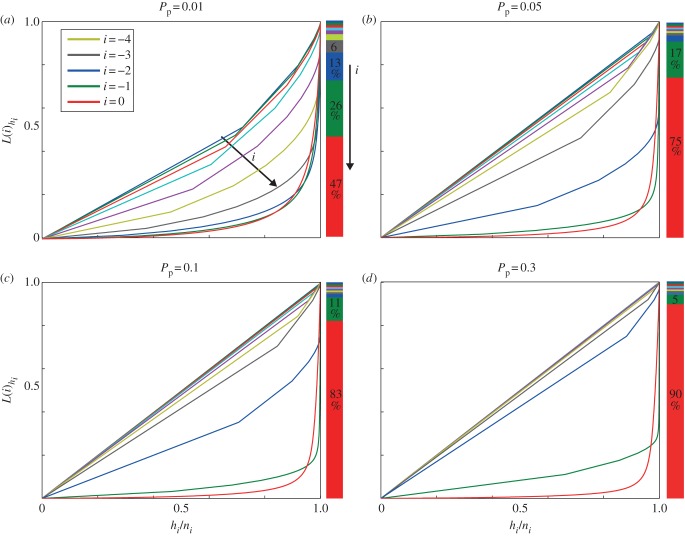
Column Lorenz curves *L*(*i*)*_h_i__* for different proliferation rates *P*_p_. The position *i* indicates the position relative to the wavefront, where *i* = 0 corresponds to the wavefront. The stacked bar chart on the right of each subfigure indicates each column’s average percentage contribution to the final population. (*a*–*d*) *P*_p_ = 0.01, 0.05, 0.1, 0.3. (Online version in colour.)

We determine the Lorenz curves for equivalent starting positions, that is those with the same-column position. The superstar dynamics in the lineage tracings of the right-most column of agents is still very pronounced for all proliferation rates (figure 9). Hence, within the right-most column, only a very small proportion of agents account for a substantial proportion of the total final number of agents. Consequently, superstar behaviour is not simply a function of agents having different initial positions, but also of stochastic competition through volume exclusion. For increasing distance from the wavefront (increasing *i*), the lineage distributions become more equal, and this occurs more rapidly as the proliferation rate increases.

The stacked bar chart in each of the subfigures in figure 9 shows us the relative size of each column subpopulation to the total population. We observe that superstars dominate in the Lorenz curve for each column that contributes a significant proportion to the final population. Furthermore, as the proliferation rate increases, the contribution to the final agent population becomes increasingly dominated by the first few columns. These contributions to the final population align with the changes in the equality of the curves, with those columns that contribute insignificant progeny to the population having an equal Lorenz curve (e.g. *i* = −3 in *P*_p_ = 0.3), whereas those with substantial contributions show asymmetric distributions.

At lower proliferation rates, motility is more dominant and a larger proportion of mixing occurs relative to proliferation, thus increasing the probability that agents behind the wavefront will later move to the wavefront and into unoccupied regions. Consequently, wherever substantial agent proliferation occurs, it happens in a stochastically competitive way, giving superstar contributors from a population of equivalent agents.

**Figure 10.**
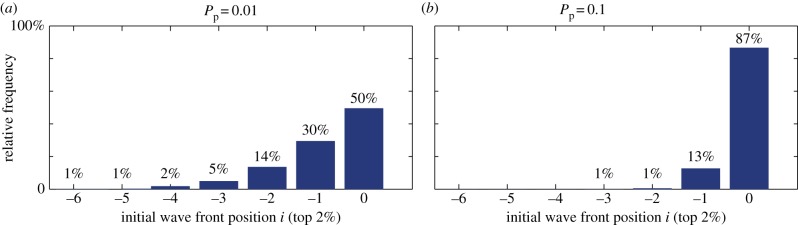
Frequency distribution of the initial position *i* relative to the wavefront of the largest 2% of lineage tracings across 200 simulations for two values of the proliferation rate. (*a*) *P*_p_ = 0.01; (*b*) *P*_p_ = 0.1. (Online version in colour.)

Figure 10 confirms these results by showing the relative frequency of the starting columns for superstar agent tracings (taken here as the largest 2% of agent lineage tracings across 200 simulations, cf. figure 8). For the lower proliferation rate (*P*_p_ = 0.01), superstars most often originate from the right-most column, but with half of them coming from behind the right-most column, whereas, when the proliferation rate is increased (*P*_p_ = 0.1), the superstars originate largely from the right-most column.

### 3.3. Comparing model results with experimental data

In a single model simulation, the lineage traces of all agents within the invasion wave are recorded at all times, whereas, in each ENC experiment, there is a single traced ENC cell, and data are collected for a single time point across numerous specimens. Therefore, in a biological experiment, each data point arises from an independent invading population, because every starting cell cannot be individually identified and uniquely labelled. By contrast, every agent can be tagged in our simulations, allowing for vastly more efficient data collection techniques. Therefore, it is appropriate to assess the differences of these related datasets in the following quantitative way.

**Figure 11.**
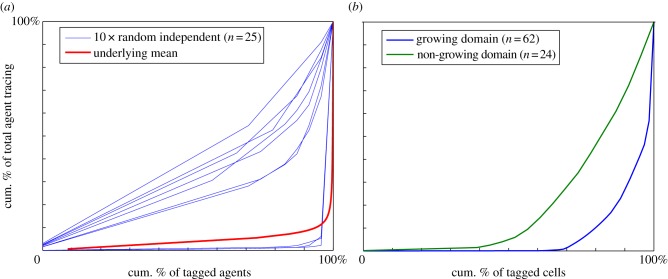
Comparison between simulation and experimental Lorenz curves. (*a*) Ten Lorenz curves (blue) from randomly sampling a single agent from *n* = 25 *in silico* non-growing experiments, *P*_p_ = 0.05 and *α* = 0. Lorenz curve (red thicker lower curve) from a single *in silico* experiment where all initial agents are tagged (indistinguishable from the one in figure 6 with same *P*_p_ and *α* values). (*b*) Lorenz curve from the experimental data (combining the wavefront and behind the wavefront data) in table 1 (no growth, blue lower curve) and table 2 (growth, green upper curve). (Online version in colour.)

At the start of each *in silico* experiment (simulation), a single agent is randomly chosen; the experiment is terminated when the total agent number reaches the required target (6524). We repeat this experiment 25 times, and in this way, we collect 25 agent lineages, giving us ranked lineage data *x_j_*, *j* = 1, 2 *…* , *n*, where *n* = 25. A single Lorenz curve is constructed from these data. This sampling process is repeated 10 times, and in this way, 10 Lorenz curves are produced, illustrated in figure 11*a*. These curves (blue) exhibit large variability, and differ greatly from the single simulation where every initial agent is traced (figure 11*a*, red curve). Even though the underlying Lorenz curve of each independent simulation is remarkably stable, the sampling from independent experiments introduces significant variability (even when the total number of agent lineages is the same). This behaviour occurs because only a small proportion of the equivalent same-column agents will be superstars. Consequently, random samples will result in a highly variable number of these superstars being chosen across independent experiments, thus introducing the variability observed in figure 11*a*, despite the population behaviour being predictable. It is worth noting that the total final number of agents is different in each of the 10 Lorenz curves, unlike our previous analysis. This increases the variability. Similar behaviour occurs for growing domain simulations.

This provides insight into the difficulty of performing detailed analysis on individual lineage tracings as given by the ENS experimental results. Furthermore, this sampling issue and the mathematical modelling provides strong motivation for undertaking experiments where the lineage of all biological cells is traced.

We calculate the Lorenz curves for the *n* experiments for the non-growing and growing gut data presented in tables 1 and 2. We see that the results for the growing gut show greater inequality than the non-growing case (figure 11*b*). Because the proliferation rates of the ENC will be the same in both cases, this result is the opposite of the result documented in figure 6*b* when all agents are tagged. This most likely occurs because of the large variability introduced by tagging a single cell in the biological experiments and also because the total final cell number differs greatly between growing and non-growing cases. This result is, indeed, consistent with figure 11*a*.

## 4. Discussion and conclusion

An agent-based model of a cell invasion process was developed to give individual agent contributions to the resulting invasion wave. These were analysed using Lorenz curves and the Gini coefficient. These econometric tools, used to measure inequality in wealth distributions, have also been used to assess plant size and fecundity [24], carbon emissions [42] and industrial planning [43]. To the best of our knowledge, this is the first application to the area of cell biology and in particular cell lineage tracings.

The agent-based model encoding movement, proliferation and gut growth provides insights into the development of the ENS. The well-described, stereotyped pattern of ENS development at the population level emerges from the local stochastic rules governing agents. However, at the same time, the individual agent lineage contributions exhibit large variability. Across all simulations a very small proportion of the agent lineages can account for a majority of the final population. This individual variability has been observed in our ENS experiments.

These results have similarities with the stochastic evolution of the epithelial cell populations of intestinal crypts to a monoclonal origin [44,45]. However, there are significant differences between the two biological systems. The achievement of local clonal dominance in a crypt occurs in a context of cell death and replacement in a non-growing domain, with non-dispersed and non-intermixed cell populations. By contrast, in the ENS, the attainment of disproportionate clonal expansion of a few clones occurs without significant cell death in a growing domain with mesenchymal ENC cells that continue to move independently and are dispersed, and which are at all times intermixed with ENS cells of different clonal origins, as well as with cells of different lineages (mesoderm cells).

The Lorenz curves for agent lineage demonstrate that the major proportion of the total population are derived from a very small proportion of the founder agents. The presence of superstars in individual simulations is robust to changes in possible experimental conditions (see the electronic supplementary material for further details). The simulation results suggest that ENS experimental superstar lineages are not ‘one offs’ or freaks, but must simply be those experiments where the large contributors just happen to be marked. A significant insight from the modelling results suggests that the data collected for the non-growing experiments have not identified a superstar. However, a superstar has been identified for the growing domain case—this was by pure chance.

The analysis highlights the difficulties of inferring cell lineage behaviour when only a single cell is tracked in each experiment (even when, as in our case, the underlying dynamics across all simulations appears to be very stable). Our analysis points to the advantage of tracing every lineage simultaneously within a biological process. Recently, progress in this direction has been possible across different experimental systems through unique inheritable imprinting [5], multi-spectral cell labelling systems [2–4,44] and advances in light microscopy and image processing [6,8]. However, at present, they are limited to a relatively small number of cells able to be marked in parallel (compared with about 8000 starting cells in this assay), and are not available for the ENS.

The basic assumptions in our modelling approach are that ENS cell movement and proliferation is stochastic within the bound of an exclusion rule, and with cell density limited to a local maximum via proliferation governed by competition for resources such as growth factors (see the electronic supplementary material). (ENS cell numbers are not regulated by competitive cell death, unlike the rest of the nervous system [32].) The experimental and modelling results presented here suggest that the stochastic competition for resources is an important concept to be considered when understanding biological processes which feature high levels of cell proliferation, especially in a developmental context. Recent advances in cell lineage tracing and computational modelling have also increased our research capacity to further explore these concepts in new systems, and in unprecedented detail, making this a new and exciting research area.

**Figure 12.**
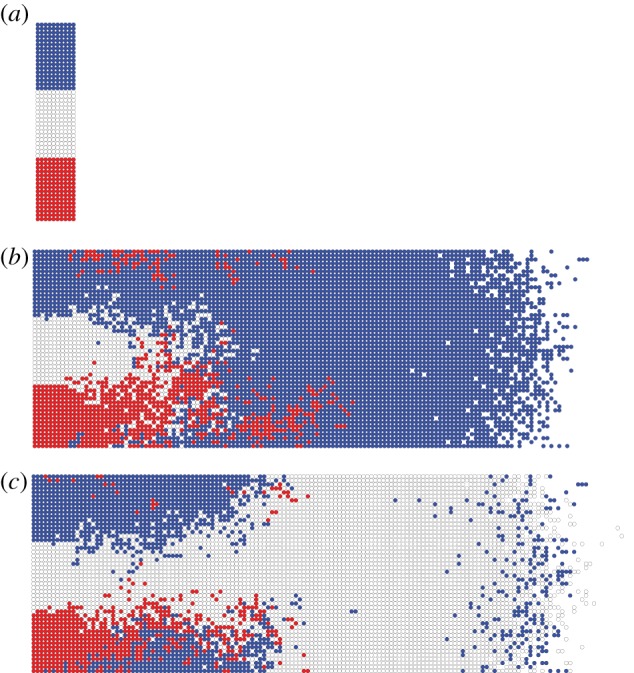
Two simulation results if agents have a predetermined fate before the invasion process commences (non-growing case). (*a*) Initial condition with three cell fates. (*b,c*) Two realizations with very different outcomes. Here, *P*_p_ = 0.05.

Our model results combined with our experimental evidence have implications for cell-fate processes in the ENS. Figure 12 shows two identical *in silico* experiments where the initial population is partitioned equally into three different groups (blue, white and red). (The growing domain case is similar and shown in the electronic supplementary material, figure S5.) This is used to represent a ‘hard-wired’ agent/cell-fate model, whereby the initial agents all have predetermined fates. The two identical *in silico* experiments show completely different final population group dynamics. This indicates that such a cell-fate control mechanism is incompatible with our model of the ENS migration process. Therefore, it seems likely that cell fate is determined after the migration wave process, and that local environment-based cell decisions at a later stage results in the highly regular cell-type proportions that are observed in developed ENS [17]. Indeed, in any developmental system with early fate decisions, tight regulation between intercellular spatial distributions and proliferation cycles would seem to be required to preserve cell-type proportions.
